# Fully integrated sampler and dilutor in an electrochemical paper-based device for glucose sensing

**DOI:** 10.1007/s00604-021-04946-3

**Published:** 2021-08-20

**Authors:** O. Amor-Gutiérrez, E. Costa-Rama, M. T. Fernández-Abedul

**Affiliations:** Departamento de Química Física y Analítica, Facultad de Química, Universidad de Oviedo, 33006 Oviedo, Spain

**Keywords:** Enzymatic biosensor, Paper-based electroanalytical device, Lab-on-paper, Sampler, Dilutor, Glucose determination

## Abstract

An electroanalytical platform capable to take and dilute the sample has been designed in order to fully integrate the different steps of the analytical process in only one device. The concept is based on the addition of glass-fiber pads for sampling and diluting to an electrochemical cell combining a paper-based working electrode with low-cost connector headers as counter and reference electrodes. In order to demonstrate the feasibility of this all-in-one platform for biosensing applications, an enzymatic sensor for glucose determination (requiring a potential as low as −0.1 V vs. gold-plated wire by using ferrocyanide as mediator) was developed. Real food samples, such as cola beverages and orange juice, have been analyzed with the bioelectroanalytical lab-on-paper platform. As a proof-of-concept, and trying to go further in the integration of steps, sucrose was successfully detected by depositing invertase in the sampling strip. This enzyme hydrolyzes sucrose into fructose and glucose, which was determined using the enzymatic biosensor. This approach opens the pathway for the development of devices applying the lab-on-paper concept, saving costs and time, and making possible to perform decentralized analysis with high accuracy.

**Graphical abstract:**

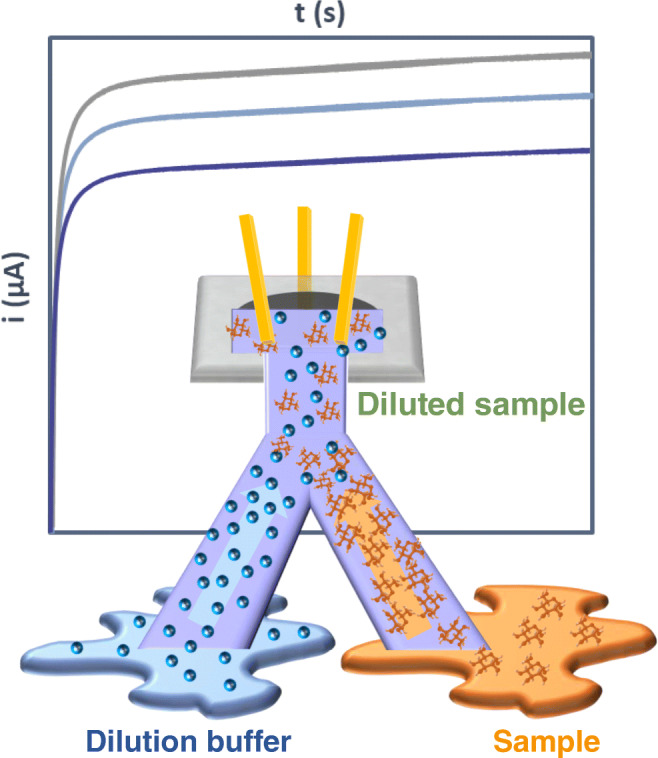

**Supplementary Information:**

The online version contains supplementary material available at 10.1007/s00604-021-04946-3.

## Introduction

Nowadays, the demand of (bio)chemical information required to make faster decisions is increasing drastically. Current trends in analytical chemistry are aimed to improve productivity-related properties, such as simplicity, rapidity, portability and cost, while maintaining sensitivity and selectivity, as well as precision and accuracy of the results. These properties imply, in most of the cases, the minimization or even the avoidance of sample preparation processes [1]. On the other hand, the objective of the well-known concept of green analytical chemistry is to reduce or even avoid the side effect of the different analytical operations both in the case of the environment and the operator [2]. In this context, the development of automated and miniaturized analytical devices that avoid an external sample treatment integrating different steps of the analytical process, saving energy and reducing the use of reagents, is among its principles [3].

Microfluidic paper-based analytical devices (μPADs) have paved the way for the development of simple, easy to use, portable, low cost, and disposable point-of-care tests. One of the characteristics that make paper an excellent substrate for microfluidic applications is its porous nature. This property enables the straightforward immobilization of reagents and the easy transport of the fluids. The capillary-driven flow does not need any pumps usually required in conventional microfluidic devices [4, 5]. However, the porosity of paper can be a double-edged sword, since liquid wicking can be slow and difficult to control [6]: under ambient conditions, paper-based (Whatman No. 1) channels, with cross-sectional dimensions of 2 mm in width and 0.2 mm in height, are able to wick liquids through distances below 3 cm in minutes, but it can take hours when long distances are needed [7]. That is the reason why, although that kind of paper is the best for electrochemical detection, as previously demonstrated by Núnez-Bajo and coworkers [8], other non-cellulosic materials (for example, those made of glass fiber) can be used for microfluidic devices.

The well-known micro total analysis system approach (μTAS) implies the integration of different analytical operations in one integrated device [9]. An ideal μTAS device should involve, apart from detection, the introduction of the sample [10] as well as other operations such as e.g. electrophoresis [11] or different biointeractions [12, 13]. Moreover, it should be easily utilized by end-users, who are not necessarily qualified personnel, so the use of laboratory stuff such as micropipettes should be avoided.

Besides that, the combination of paper-based microfluidics with electrochemical detection allows the development of sensitive miniaturized devices for interesting applications. Moreover, the fast advance in the “internet of things” makes possible the existence of portable and interconnected electronic readers for on-site measurements that can share information [14]. Nowadays, available portable potentiostats, with very different designs, are able to perform almost every electrochemical technique [15]. Because of this and the simplicity and good analytical features of electrochemical techniques, paper-based electroanalytical devices have a great potential to construct devices for rapid and decentralized analysis for many practical applications [16].

In this work, trying to go one step further in the lab-on-paper concept and the requirements of green analytical chemistry, advances have been made on the automation of methods and the integration of different steps of the analytical process in one platform. This challenge focuses on incorporating an accessory able to dilute the sample at the same time it is transported from the container to the electrode. In this context, a three-dimensional microfluidic “dilutor” device has been designed using glass-fiber pads, commonly used in lateral flow immunoassays. This pretreatment of the sample is usually required to dilute the sample until it is within the useful concentration range of the methodology, and also to have the sample in the medium adequate for the measurement (optimum conditions of e.g. pH, ionic strength, etc.). To the best of our knowledge, this is the first time that a glass-fiber pad is used as sampler and dilutor simultaneously for the development of electrochemical lab-on-paper devices. Different strategies have been carried out in order to design paper-based devices able to mix reagents or even dilute, using nitrocellulose membranes [17, 18] or stacking different layers of paper [19]. However, these operations are usually performed offline using micropipettes to dilute and deposit later the diluted sample on the device [20, 21], or designing microfluidic systems, delimiting different channels with hydrophobic barriers [11, 12]. In this case, the novelty resides in the fact that dilution is performed by coupling another simple glass-fiber strip to the sampler, both made by cutting. Dimensions could be varied depending on the concentration of the sample.

Glucose has been commonly used as model analyte for the development of different sensing approaches due to its high importance in biochemical functions. Moreover, glucose determination is also essential in food analysis. In many food products, the concentration of glucose is very high, and, therefore, samples often should be diluted before analysis. Thus, glucose was chosen as model analyte to demonstrate the suitability of this platform to develop an integrated glucose enzymatic biosensor.

## Materials and methods

### Chemicals

Glucose oxidase (GOx), horseradish peroxidase (HRP), invertase, potassium ferrocyanide, *N,N*-dimethylformamide, Trizma® base (2-amino-2-(hydroxymethyl)-1,3-propanediol) and the Glucose Assay Kit were provided by Sigma-Aldrich. Enzymes were stored in the freezer (at −20 ^o^C), and they remain outside only while weighing. Anhydrous D-(+)-glucose was purchased from Merck. D-(+)-sucrose was purchased from VWR International. Carbon ink was provided by Gwent Electronic Materials Ltd. All chemical reagents were of analytical reagent grade and were used as received without further purification. Ultrapure water obtained from a Millipore Milli-Q purification system (Millipore Direct-Q™ 5) was used throughout this work. Glucose stock solutions were prepared daily in a 0.1 M Tris-HNO_3_ pH 7.0 buffer solution. This buffer and the solutions of GOx, HRP and invertase (in Tris-HNO_3_ buffer solution) were prepared weekly and stored at 4 °C.

### Apparatus and measurements

Measurements were performed using a μAutolab type II potentiostat/galvanostat from Eco Chemie interfaced to a computer system controlled by the NOVA 2.1 software from Metrohm Autolab. A connector (ref. DRP-DSC) purchased from DropSens was used as an interface between the paper-based platform and the potentiostat to perform the measurements. Whatman™ paper (100×300 mm) and a wax printer XEROX ColorQube 8570 were used for the fabrication of paper-based electrodes. Gold-plated connector headers were delivered by Digikey. Glass-fiber sample pads were purchased from Merck Millipore. Transparency film was purchased from Stabilo.

### Electrochemical cells

The electrochemical cell used in this work consisted of a paper-based working electrode (WE) and a gold-plated connector header with three pins as reference (RE) and auxiliary (AE) electrodes, and also as connection between the working electrode and the potentiostat. The procedure to construct this electrochemical cell, described in detail in previous works [10, 22], was as follows: The working hydrophobic area was patterned with wax, which was diffused with heat at 100 °C for 1 min. Then, carbon ink (40% in DMF) was deposited as previously described. The paper layer containing the working electrode (with 4 mm in diameter and 12.6 mm^2^ in area) was placed between the pins of the gold-plated connector headers, with one at the bottom (the connection between WE and the potentiostat) and the other two (RE and AE) on the top. A picture of the materials required and the experimental setup is shown in Fig. S1.

### Sampler/dilutor integration

**Fig. 1:**
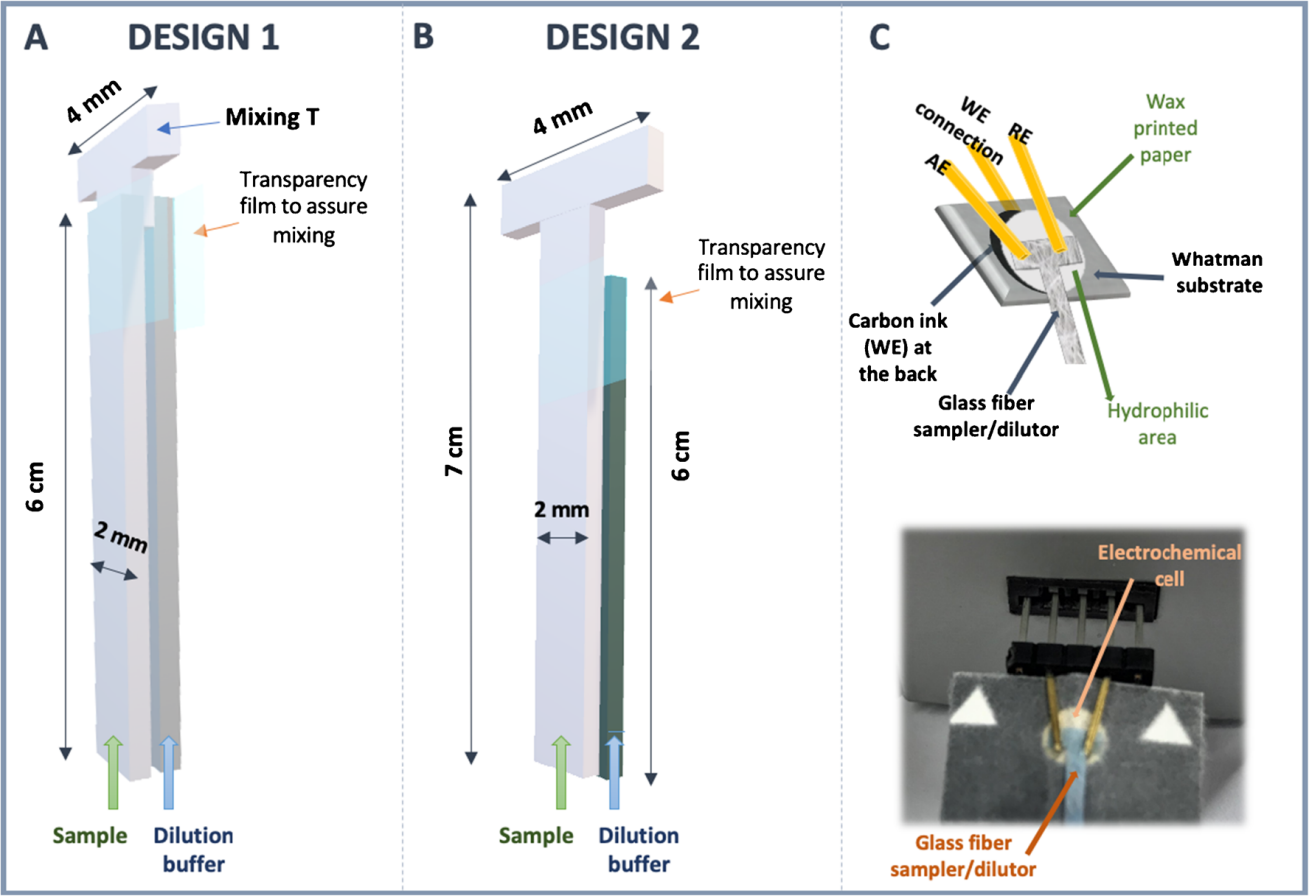
Schemes of the two designs developed in this work: (**A**) design 1, which uses two straight strips and one short “T” piece for mixing, and (**B**) design 2, using a long “T”-shaped sampler and a straight strip for dilution. (**C**) Scheme (top) and picture (bottom) of the assembled microfluidic paper-based device including gold-plated headers

The piece acting as dilutor was made of a glass-fiber conjugate pad and was combined with the paper-based working electrode. Two designs have been assessed. The first one (design 1) consisted of three pieces, two long strips and a shorter one which is “T” shaped. The stem of the “T” was placed between the two long strips to receive both fluids (sample and dilution buffer) and mix them (Fig. 1A). The three layers were stacked inside a folded transparency film that were held together with adhesive tape at the ends, to avoid leaking through the transparency film. After that, the piece of the “T” located out of the film was placed over the paper-based electrode (by the side opposite to the carbon ink) and was clipped with the connector headers, converting it into a robust platform. The upper part of the “T”-shaped mixing pad was 4 mm wide (as the diameter of the WE) and 2 mm long. The stem of the “T” was 10 mm long and was placed between two long strips (2 mm wide and 6 cm long). This length can be varied depending on the distance to the containers.

The second design (design 2) is a simpler version since it only requires two strips (Fig. 1B): one, the longest (7 cm long and 2 mm wide), with a T-shape end is immersed in the sample container (sample strip); the other, 1 cm shorter, is immersed in the dilution buffer (dilution strip). The width of this last strip can equal that of the T-shape strip or be greater if a higher dilution is desired. In this case, the strips were also stacked inside a folded transparency film.

In order to dilute and analyse samples, one strip was put in contact with dilution buffer, and the other one with the sample solution, allowing them to rise by capillarity and mix in the part where the transparency film was pressed to allow the mixing. Finally, the diluted solution arrived to the upper part of the “T” and, consequently, to the electrode, wetting the circular working area and contacting the AE and RE, as seen in Fig. 1B. The dilution device was positioned on the center of the electrochemical cell, over the paper-based electrode, and the “T” was clipped between the gold-plated connector headers and the paper-based electrode, to guarantee the ionic contact between the solution and the electrodes, allowing suitable ionic connection. A scheme of the assembled device, which includes the microfluidic platform and the paper-based electrochemical cell can be observed in Fig. 1C.

### Electrode (bio)modification

The concentration of the enzymes (GOx and HRP) and the mediator, ferrocyanide, was previously optimized [22]. Thus, a volume of 5 μL of a mixture of enzymes and mediator (with 1.6 U·μL^−1^ of GOx, 2.5 U·μL^−1^ of HRP and 0.1 M of ferrocyanide) was prepared in 0.1 M Tris-HNO_3_ buffer solution pH 7.0 and dropped over the paper-based working electrode, at the opposite side of the carbon ink. It was left to dry for approximately 30 min. Biosensors were used at room temperature.

### Strip modification with invertase

A volume of 20 μL of an invertase solution of 1 U·μL^−1^, prepared in 0.1 M Tris-HNO_3_ buffer solution pH 7.0, was dropped in the middle of the sampler strip and left to dry for approximately 30 min.

### Electrochemical measurements

In order to perform the measurements, the pins at the rear part of the connector were inserted in the commercial interface to connect to the potentiostat. Once the experimental setup was ready, as shown in Fig. S1, the general procedure was as follows: Liquid sample was directly taken from a 1.5-mL container by introducing the sample strip. Then, it was mixed with the dilution buffer, equally taken from another 1.5-mL container (in both cases ca. 50 μL·cm^-2^). Samples do not need any treatment apart from dilution, performed with the dilutor. In any case, the porous material could act as a filter for particles of specific size. Regarding the buffer, its composition depends on the application, in this case 0.1 M Tris-HNO_3_ pH 7.0. To know the adequate dimensions of the dilution strip, it is necessary to estimate the approximate glucose concentration in the sample, to be within its calibration range. This information could be extracted from the food label/package. When the diluted sample fills the pores of the electrochemical cell containing immobilized enzymes, the electrochemical measurement (either cyclic voltammogram or chronoamperogram) can be performed.

Then, measurements to assess the features of the dilutor were performed as follows: different concentrations of ferrocyanide were used and, once the solution arrived at the electrode after being diluted with buffer solution, cyclic voltammograms (CVs) were recorded scanning the potential from −0.2 to +0.5 V at a scan rate of 50 mV·s^−1^.

When measuring glucose, chronoamperograms were recorded applying a potential of −0.1 V (vs. gold-plated wire) for 50 s, using as analytical signal the current measured at that time, which is due to the reduction of the ferricyanide previously generated by the enzymatic reaction on the surface of the modified working electrode [10, 22]. This current is directly related to the concentration of glucose.

The paper-based electrode and microfluidic sampler/dilutor were used only once, but the connector header could be reused for repeated times, after a washing step with water, without affecting the signals.

## Results and discussion

Advancing on the full integration of steps of the analytical process in one single device, in this work, a sampling and diluting platform is integrated with a paper-based transducer that fits perfectly with commercial interfaces. This device avoids the use of micropipettes, apart that for sampling, for simple pretreatments such as dilution. It has been fabricated taking advantage of a microfluidic piece previously reported [10] in such a way that, after redesigning and including add-ons, a new operation can be performed in a very easy way.

### Electrochemical feasibility and dilution ability of the integrated platform

To test the performance of the microfluidic sampler/dilutor platforms, a well-known electroactive compound such as potassium ferrocyanide was used. A cyclic voltammogram recorded when a drop of a 10 mM ferrocyanide solution was directly deposited (offline) on the hydrophilic area of the electrochemical cell. The redox process, characteristic of the ferro/ferri system, was clearly observed (dark gray continuous and striped lines in Fig. S2A, B). Then, after coupling a new paper-based electrochemical cell with the design-1 sampler/dilutor (Fig. 1A), CVs were similarly recorded. As can be seen in Fig. S2A, when Tris-HNO_3_ buffer was absorbed through both channels, no redox process was observed, but when one strip was immersed in Tris-HNO_3_ buffer and the other one in a 10 mM ferrocyanide solution, the orange line showed the signal of the ferro/ferri system, confirming the dilution of ferrocyanide. Using design 2 (Fig. 1B), the CVs obtained were homologous, as seen in Fig. S2B, but, in this case, the dilution was lower (44% with design 2 against 70% with design 1).

As can be seen in Fig. S2, there was a clear dilution when strips were immersed in a ferrocyanide solution and in a buffer solution, as shown by the decrease in peak current intensities, for both oxidation and reduction, compared to those obtained when a drop of ferrocyanide was deposited on the WE. This suggests the ability of our one-dimensional microfluidic add-on to dilute a sample while transporting it to the electrode where it was detected, with both designs. However, the second design was simpler to prepare since only two components were needed (the sampler “T”-shaped strip and the dilutor strip), and moreover, both operations occurred faster (30 s against 1 min for design 1). Therefore, this design was employed for further studies.

**Fig. 2:**
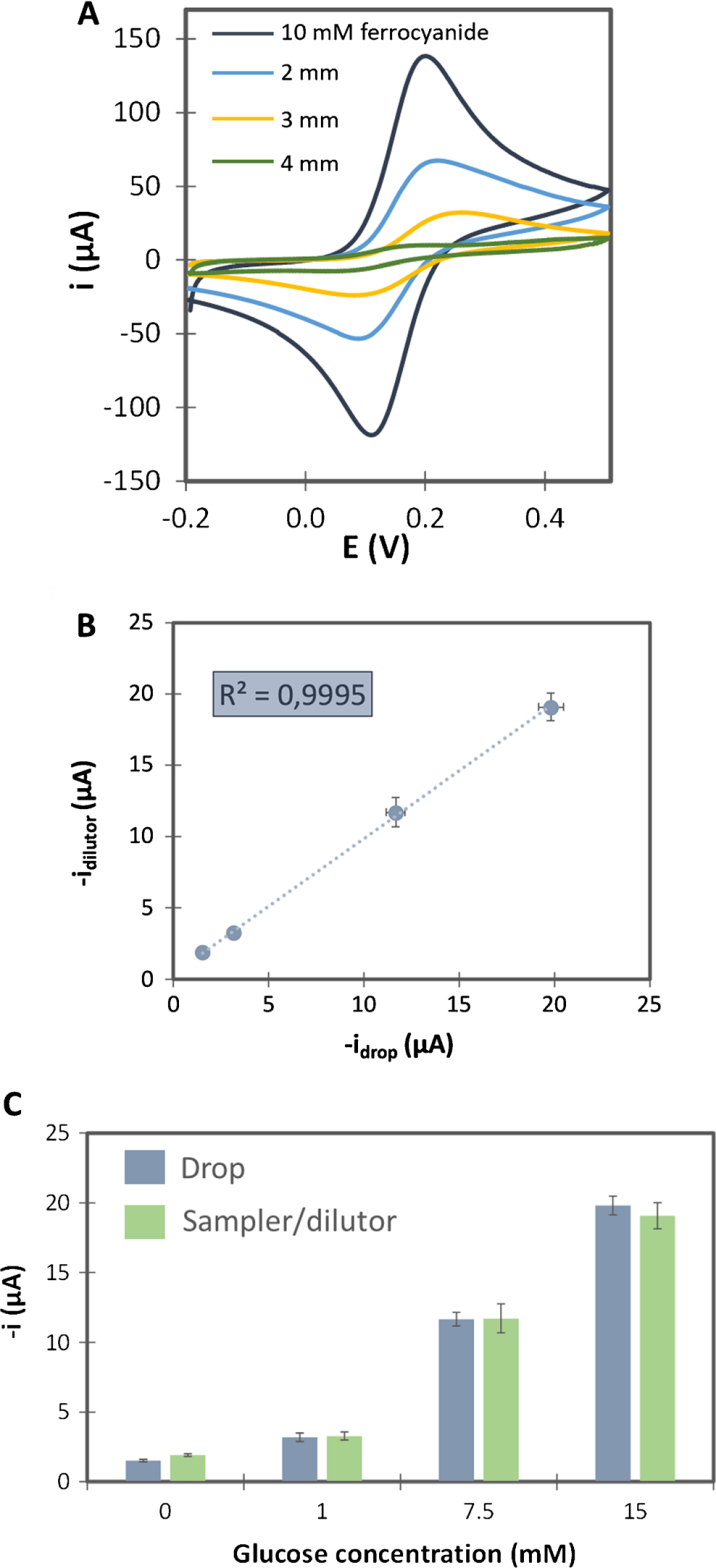
**A**) CVs obtained using different widths (2, 3 and 4 mm) of the dilution strip (design 1) immersed in buffer solution. The sample strip was always immersed in a 10 mM ferrocyanide solution. Signals were compared with this obtained from a CV recorded when a drop of 10 mM of ferrocyanide was directly deposited on the WE. CVs were recorded at a scan rate of 50 mV·s^−1^. **B**) Comparison of current intensities obtained for glucose concentrations after depositing drops directly on the electrochemical cell and after dilution of  20-times-higher glucose concentrations, measured with the developed platform (design 2). **C**) Bar diagram of the current intensities obtained for glucose concentrations after depositing drops directly on the electrochemical cell (blue bars) and for 20-times-higher glucose concentrations measured after dilution with the developed platform (green bars). Chronoamperograms were recorded applying −0.1 V (vs. gold-plated wire) during 50 s. Data are given as average ± SD (*n*=5)

Once proved the dilution ability of the two-strip platform and considering that samples with different ranges of concentrations would need different dilution ratio, strips with different widths (2, 3 and 4 mm) were tested (maintaining the same width for the “T”-shaped strip). The results of this experiment can be observed in Fig. 2A that compares the signals with that obtained for a drop of a 10 mM ferrocyanide solution.

As can be seen, when increasing the width of the dilution strip, the signal decreases accordingly, indicating that the dilution factor raises by enlarging the width of the dilution strip. To extract quantitative data from Fig. 2A a calibration graph has to be constructed first by depositing directly drops of increasing concentrations of ferrocyanide and representing the anodic peak current of the CVs recorded vs. the concentration of ferrocyanide (see Fig. S3). Table 1 summarizes the dilution ratio obtained for each width, after obtaining the value of the ferrocyanide concentration in the electrochemical cell using the “offline” calibration curve.

**Table 1:** Dilution ratios obtained using dilution strips (design 2) with different widths that were immersed into a 10 mM ferrocyanide solution. Data are given as average ± SD (*n*=5)

Width (mm)	Concentration in the container (mM)	Anodic peak current intensity (μA)	Concentration in the cell (obtained from the calibration plot)	% RSD	Dilution
2	10	59 ± 4	5.6 ± 0.5	9.3	**1:1.8**
3	10	22 ± 2	2.2 ± 0.2	6.9	**1:4.6**
4	10	4.46 ± 0.09	0.49 ± 0.01	1.7	**1:20**

As can be seen in Table 1, when increasing the width of the dilution strip, higher dilution was achieved, and the precision improved simultaneously. This can be due to the better precision in the fabrication and therefore in the strip dimensions. Dilutions of approximately 1:2, 1:5 and 1:20, for of 2, 3 or 4-mm-wide dilution strips, were achieved respectively, showing the applicability of this platform for the analysis of different samples that require different dilution factors before analytical signals were recorded.

### Glucose measurements

In order to demonstrate the feasibility of this platform, the sample and dilution strips were combined with the electrochemical cell modified with the enzymes (GOx and HRP) and the mediator (ferrocyanide) and applied to the quantification of glucose in different solutions. The analytical signal corresponds to the electrochemical reduction of the ferricyanide, obtained after the enzymatic reactions. Previously, a calibration plot of glucose was performed depositing directly a 10-μL drop of glucose solution on the electrochemical cell (Fig. S4). Then, using the sampler and the 4-mm dilution strip (that provides a 1:20 dilution factor, required for glucose analysis in samples such as orange juice and a cola beverage) [22], signals for glucose concentrations of 20, 150 and 300 mM were recorded by chronoamperometry (measured at −0.1 V vs. gold-plated wire) (Fig. 2B,C). Those glucose concentrations were chosen since, when diluted 1:20, they correspond to 1.0, 7.5 and 15 mM concentrations, i.e. at low, medium and high level of concentration of the calibration graph shown in Fig. S4.

As seen in Fig. 2B,C, the intensities obtained with the dilutor corresponded to those obtained when 20 times lower glucose concentrations were measured depositing drops directly on the electrochemical cell. These results bring out that the dilutor showed a good accuracy regarding the dilution factor obtained. The reproducibility was also very good, with relative standard deviations (RSD) below 9.6% for all the measurements performed.

The integration of the dilution and sampler strip with the sensor provides a reliable platform able to perform several steps (sampling, dilution, coupled reactions, detection) required for the analysis without the need of micropipettes, microtubes or similar laboratory materials. Moreover, the dilution strip makes possible the glucose determination in a very wide concentration range when compared with other glucose sensors recently reported (see Table S1). Regarding storage lifetime of the device, although in this work stability studies have not been included, it is clear that the most critical is due to the immobilized enzymes, and this has been studied in a previous work [22] demonstrating that the platform maintains the initial analytical signal at least for 1 week (when it is stored at 4 °C protected from the light). In any case, if commercialization is aimed, a more thorough study should be made.

### Real sample analysis

After testing that the dilutor worked adequately for the enzymatic determination of glucose, real food samples like a commercial orange juice and a cola beverage were analyzed in order to quantify glucose concentration. The only sample pretreatment needed was stirring of the cola beverage in order to remove the gas. After that, the developed platform was used to measure the glucose concentration in both samples using the 4-mm-wide dilution strip, since it was the width able to provide the required dilution factor of 1:20. The results obtained, which can be seen in Table 2, were compared to the ones obtained with a commercially available enzymatic kit with spectrophotometric detection.

**Table 2:** Comparison of results obtained for real food sample analysis, with the sampler and dilutor platform ensembled with the paper-based glucose biosensor and with a spectrophotometric enzymatic kit. Data are given as average ± SD (*n* = 3)

Sample	Sampler and dilutor paper-based enzymatic biosensor	Spectrophotometric assay
Cola beverage	3.1 ± 0.4 g/100 mL	3.12 ± 0.03 g/100 mL
Orange juice	2.6 ± 0.3 g/100 mL	2.72 ± 0.04 g/100 mL

The concentration values were compared using the Student’s *t* test [23]: the calculated *t* values obtained for the samples analyzed with the diluting platform here developed were lower than the tabulated *t* value with two degrees of freedom and a 0.05 significance level. Therefore, we can conclude that there were no significant differences between the values obtained with both methodologies, demonstrating the accuracy of the methodology employing the sampler and dilutor platform here developed. In this context, we can also conclude that there were no significant interferences in real food matrices. The use of a potential as low as −0.1 V has two important advantages: i) it produces reduction processes, which are not as common as those required for oxidations. Many molecules of interest (drugs, pesticides…), contain amine or hydroxyl groups that are oxidized and, in most of the cases a cathodic process appears, this only happens if they have been oxidized first, ii) the value of the reduction potential is not high and then, even for molecules that could be reduced, the energy is not high enough to produce the cathodic process. In any case, the selectivity of this glucose biosensor has been studied in a previous work in which the concentration of the sensor phase was optimized [22]. In that work, mixtures of glucose with fructose and ascorbic acid (which are the most common interferences present in food containing high glucose levels) have been measured with the glucose biosensor, observing that they were not significant.

### Sucrose measurements

**Fig. 3:**
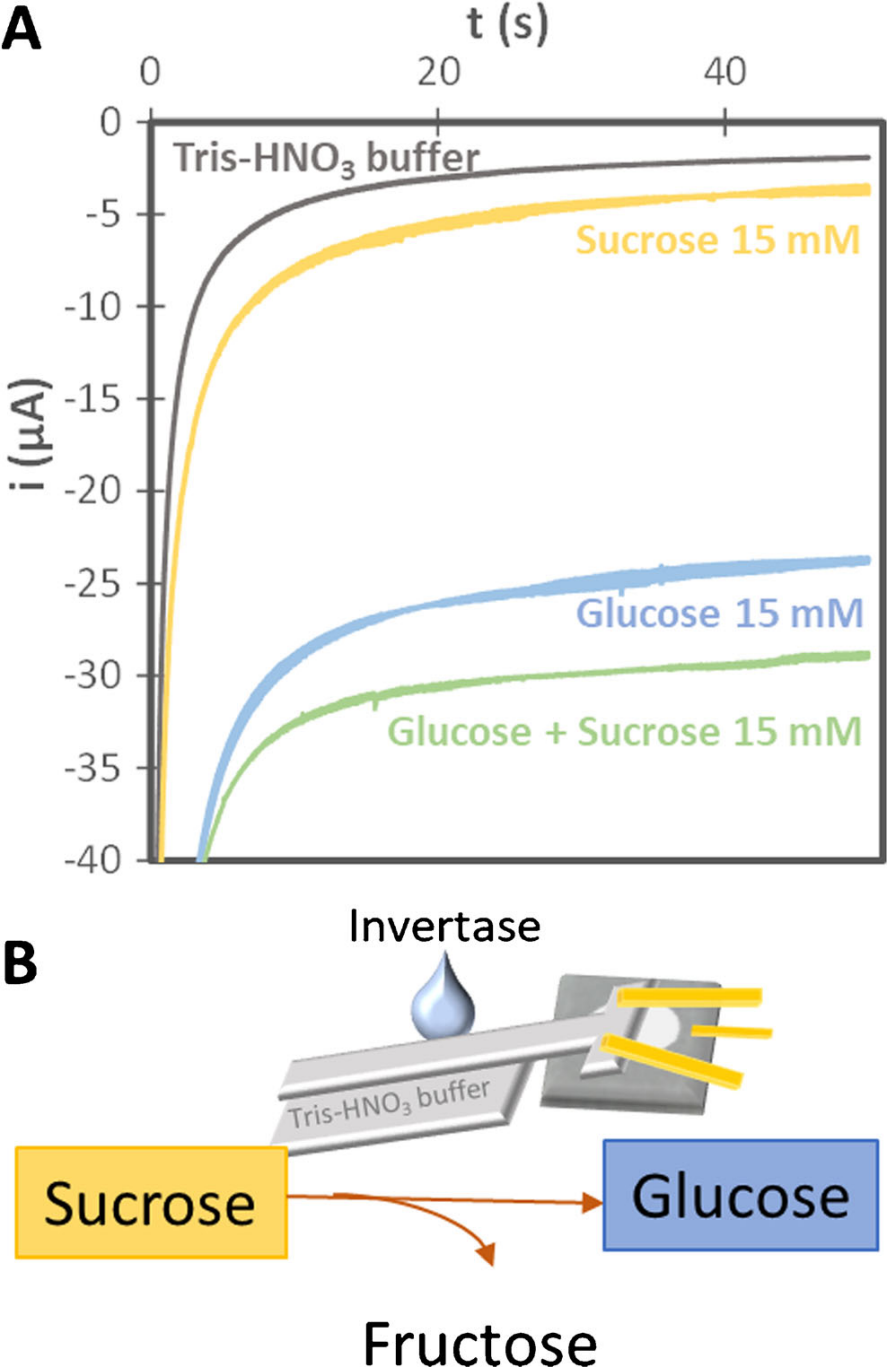
**A**) Chronoamperograms recorded with the sampling (modified with invertase) and diluting platform. Chronoamperograms were recorded applying −0.1 V (vs. gold-plated wire) for 50 s. **B**) Scheme of the enzymatic reaction produced for sucrose hydrolysis

As a proof-of-concept, and in order to move one step forward in the development of “lab-on-paper” devices, the enzyme invertase, who hydrolyses sucrose into fructose and glucose, was deposited on the sampling strip to see if the determination of both analytes in the same sample was possible. In this way, auxiliary enzymatic interactions can be also added to the integrated platforms. With this aim, invertase was immobilized in the middle of the sampling strip (in a zone that does not contact the sample) in order to avoid contaminating the sample when the strip was immersed in the container. When invertase was immobilized on the strip, sucrose was hydrolysed, and the generated glucose arrived to the electrochemical cell initiating the cascade of enzymatic reactions to produce a reduction current, whose intensity was proportional to the concentration of glucose (scheme in Fig. 3B). If there was glucose in the sample, this intensity corresponded to the total glucose concentration (native glucose plus glucose derived from sucrose), in such a way that the difference with/without invertase would allow to obtain both glucose and sucrose concentrations. The results of this experiment can be seen in Fig. 3A. It shows the chronoamperograms recorded immersing the sampling strip in Tris-HNO_3_ buffer, in a 15 mM glucose solution, in a 15 mM sucrose solution and in a mixture of 15 mM glucose and 15 mM sucrose. The diluting strip was always immersed in Tris-HNO_3_ buffer.

As can be seen in Fig. 3A, when sucrose was aspirated with the sampler, an analytical signal slightly higher than the one obtained with buffer was obtained, demonstrating the interaction of sucrose with the invertase deposited on the sampling strip. Moreover, there was also a difference between the intensity obtained with just glucose (15 mM) and with a mixture of glucose and sucrose (15 mM in each), corroborating that the invertase reacted, in some extent, with sucrose. It is important to highlight that the intensity obtained with non-inverted sucrose does not represent any interference in the glucose biosensor, since the signal for sucrose directly dropped on the glucose biosensor (without invertase) was −1.6 ± 0.3 μA (similar to the background obtained with buffer solution, which was around 1.3 μA). This paves the way to a more exhaustive work in which invertase concentration has to be optimized as well as the design including heating platforms or other add-ons to have suitable conditions (pH 4.5 and 55 °C) for reaching the highest activity of invertase.

These findings indicate that this lab-on-paper approach is able to perform different steps of the analytical process in one device, which are: i) taking the sample from the container; ii) performing reactions such as the hydrolysis of sucrose with invertase; iii) diluting the sample appropriately by using the adequate dilutor width; and iv) performing the electrochemical measurement with the help of a potentiostat, which, as commented before, could also be portable in order to achieve real point-of-use devices able to perform all-in-one, on-site measurements without the need of qualified personnel. Apart from that, the analysis of cost performed (see Supplementary Information -Table S2-) also indicated that the device is extremely cheap: it costs less than $ 0.56, taking into account that the most expensive component is the gold connector ($ 0.3), but it can be reused several times, and also enzymes. According to the time, the assembling of one device and the measuring take less than 32 min, and this time could be reduced if several microfluidic platforms were mass-produced or, even more, if a multiplexed platform was used coupled to a multichannel potentiostat [10] (Table S3).

## Conclusions

In this work, we have exploited the “lab-on-paper” approach by designing a platform able to perform different steps of the analytical process in a short time and using low-cost materials. It has been proved through the determination of glucose in real food samples, such as cola beverage and orange juice, demonstrating a very good accuracy and reproducibility. Although no treatment is usually required for glucose determination in blood samples, where peripheral blood contacts directly the platform, food samples usually contain a high amount of glucose and dilution is required to be within the detection range. This can be made easily by just adapting the width of the dilution strip. In any case, since normal glucose concentration in blood is comprised between 4.4 and 6.6 mM (being higher for diabetes mellitus patients) and the calibration range of this sensor is comprised between 0.5 and 15 mM, a single sampler could be employed. Possible matrix effects should be considered. Finally, as a proof-of-concept, invertase has been deposited on the sampling strip to perform auxiliary enzymatic reactions in the same device, for the determination of sucrose, another sugar very important in food quality control. This could also be applied to the determination of other analytes in which dilution and/or another sample pretreatment are usually required. The trends of analytical chemistry such as miniaturization, automation, reduction of costs and simplification are all achieved in this work, obtaining promising results for the development of biosensing platforms for real decentralized analysis.

## Supplementary information

ESM 1 (DOCX 1.59 mb)
