# Supplementary material for: Fully integrated sampler and dilutor in an electrochemical paper-based device for glucose sensing
Source: Mikrochim Acta. 2021 Aug 20;188(9):302. doi: 10.1007/s00604-021-04946-3 (PMC8379134; doi:10.1007/s00604-021-04946-3)
Supplement: Supplementary file 1 — (DOCX 1.59 mb) [file 604_2021_4946_MOESM1_ESM.docx]

**Electronic Supplementary Material**

**Fully integrated sampler and dilutor in an electrochemical paper-based device for glucose sensing**

*O. Amor-Gutiérrez, E. Costa-Rama, M. T. Fernández-Abedul**

*Departamento de Química Física y Analítica, Facultad de Química, Universidad de Oviedo, 33006 Oviedo, Spain*

**e-mail:* [*mtfernandeza@uniovi.es*](mailto:mtfernandeza@uniovi.es)

**Tel.: +34 985 10 29 68*

*Orcid:* [0000-0003-3917-4713](https://orcid.org/0000-0003-3917-4713) *(OAG),* [0000-0002-5357-9269](https://orcid.org/0000-0002-5357-9269) *(ECR),* [0000-0003-3782-5025](https://orcid.org/0000-0003-3782-5025) *(MTFA)*


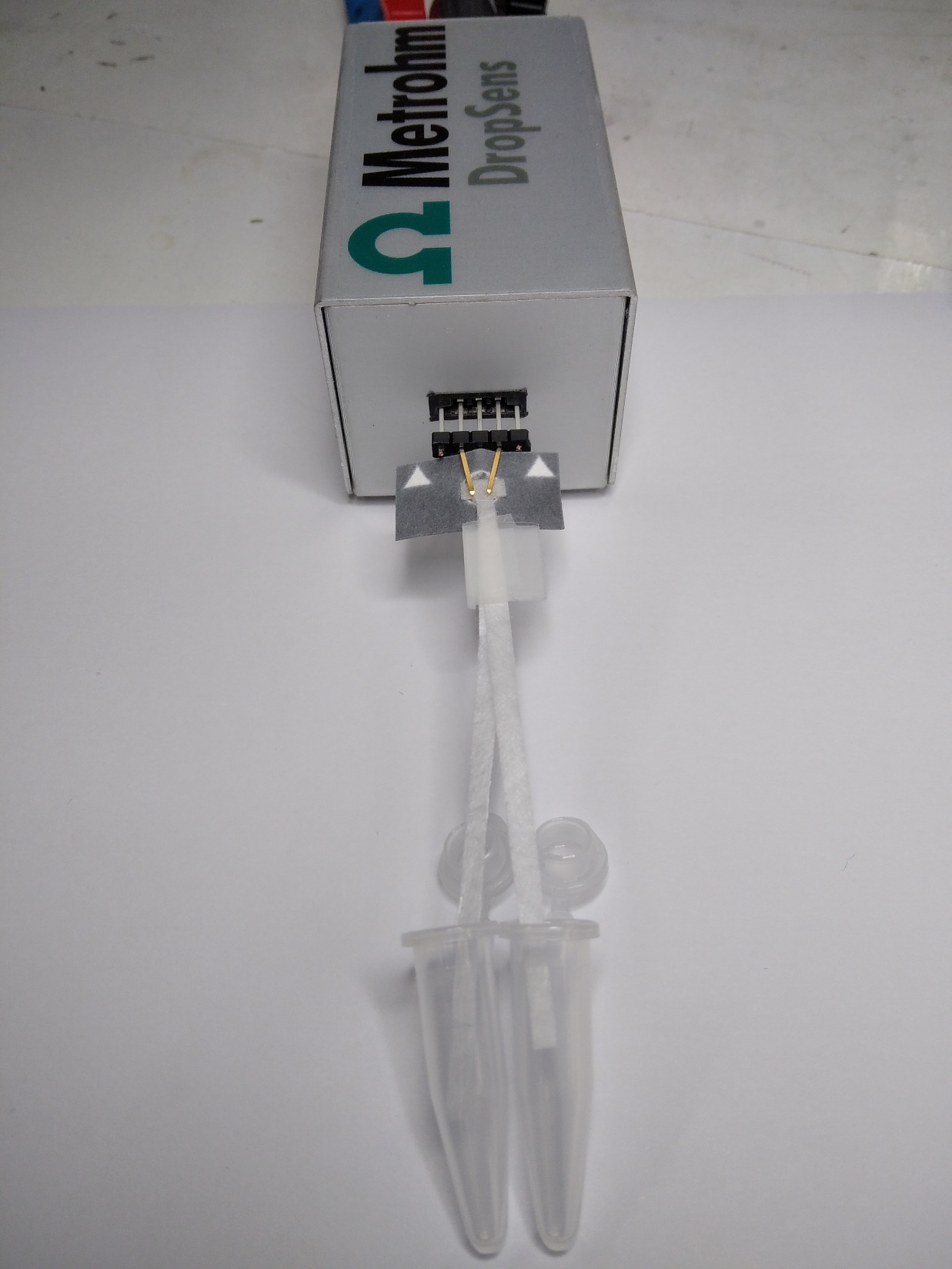

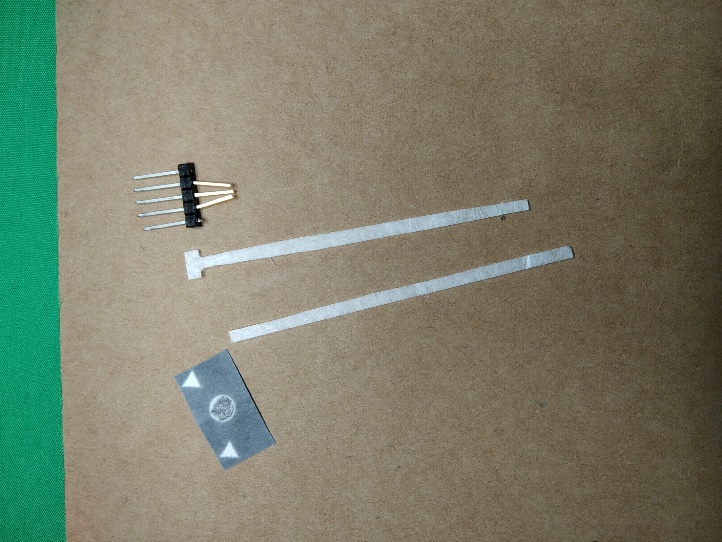


**A**

**B**

Paper-based working electrode

Dilution strip

Buffer container

Connections, reference and counter electrodes

Sample strip

Sample container

Mixing region

**Fig. S1** Picture of the A) experimental setup including sampler and dilutor for electrochemical enzyme assay and B) the materials required.

**Fig. S2** CVs recorded with the (A) design 1 and (B) design 2 using either buffer solution in both strips, or the ferrocyanide solution in the sample strip and the buffer solution in the dilution strip. For the sake of comparison, the signal recorded for the ferrocyanide without the diluting platform (adding a drop) has been also included. CVs were recorded at a scan rate of 50 mV·s^-1^.

**Fig. S3** Calibration plot obtained with the paper-based electrochemical cell without dilutor depositing a 10-µL drop of increasing concentrations of ferrocyanide, from 0.01 to 10 mM, measuring the oxidation intensities. CVs recorded from -0.2 V to +0.5 V (*vs.* gold-plated wire), at a scan rate of 50 mV·s^-1^. Data are given as average ± SD (n=5).

**Fig. S4.** Calibration plot obtained with the paper-based glucose biosensor without dilutor depositing a 10-µL drop of increasing concentrations of glucose, from 0.5 to 15 mM. The analytical signal is the current intensity obtained by chronoamperometry applying -0.1 V (*vs.* gold-plated wire) for 50 s. Data are given as average ± SD (n=5).

**Table S1.** Comparison of the analytical performance of this glucose biosensor with others found in the literature.

| **Glucose biosensor** | **Linear range (mM)** | **Steps integrated** | **Construction of the electrodes** | **Architecture of the paper-based platform** | **Analysis time (s)** | **Measurement** | **Reference** |
| --- | --- | --- | --- | --- | --- | --- | --- |
| **Paper-based biosensor with low-cost connector headers and sampling and dilution platforms** | 0.5 – 300 | Sampling, dilution, coupled reactions, detection | Stencil free, drop casting | Multilayer | 80 | Chronoamperometry | **Current work** |
| **Drawn origami paper for electrochemical detection of glucose** | 1 – 12 | Detection | Pencil drawing | Origami | 50 | Chronoamperometry | [1] |
| **Paper-based synthesis of Prussian Blue nanoparticles** | 0 – 25 | Synthesis of Prussian Blue nanoparticles, detection | Screen-printing | 1D | 60 | Chronoamperometry | [2] |
| **Screen-printed paper-based transducers** | 0.50 – 50 | Dilution, detection | Screen-printing | 1D | 30 | Coulometric detection | [3] |
| **Glucose sensor based on a fiber paper-based screen-printed carbon electrode** | 3.7 – 13.8 | Detection | Screen-printing | Multilayer | 30 | Chronoamperometry | [4] |
| **Three-dimensional paper-based microfluidic electrochemical wearable devices** | 0 – 1.9 | Sweat collection, evaporation, detection | Screen-printing | Origami | - | Amperometry | [5] |
| **3D paper-based microfluidic electrochemical glucose biosensor** | 0.10 – 25 | Detection | Photolithography | Multilayer | 200 | Chronoamperometry | [6] |

**Analysis cost and time**

Nowadays characteristics related to productivity, such as low cost and short analysis time, are very important when talking about decentralized analysis, making easier the availability of the devices especially in developing countries. The cost analysis for a sampling and diluting glucose biosensor which uses paper-based working electrodes and gold-plated connector headers is shown in **Table S2**. Taking into account the calculations done, the cost of developing a sampling and diluting glucose biosensor is less than $ 0.56, considering the quantities used of materials and reagents. The most expensive components of the biosensor are the enzymes, which can be prepared once a week in order to save costs; and the gold-plated connector headers: it is $ 0.3 per device, which is half of the total price, but it can be reused several times without affecting the analytical signals.

**Table S2.** Cost of fabrication of one paper-based microfluidic platform.

| **Item** | **Cost** | **Cost per device** |
| --- | --- | --- |
| **Whatman™ paper** | $ 0.50 / sheet (300 cm^2^) | < $ 0.003 |
| **Conjugate fiber pads** | $ 61.60 / 100 m | $ 0.037 |
| **Carbon paste** | $ 32 / 50 g | < $ 0.0003 |
| **Gold-plated connector headers** | $ 3.7 / 36 pin | $ 0.3 |
| **DMF** | $ 96 / L | < $ 0.0002 |
| **GOx** | $ 892.40 / g | < $ 0.09 |
| **HRP** | $ 311.20 / 50 mg | $ 0.12 |
| **Ferrocyanide** | $ 34.10 / 5 g | < $ 0.003 |
| **Total cost per device** | | **< $ 0.56** |

According to the analysis time, it has been checked how long the different steps take for the determination of glucose with this sampling and diluting platform, and it is shown in **Table S3**. The time needed for each sample is approximately 32 min, but it can be reduced if biosensors are mass-modified with enzymes. Apart from that, time could be even more reduced if a multiplexed platform, coupled to a multichannel potentiostat, is used, being able to perform 8 measurements at the same time. On the other hand, the methodology has shown to be very robust, so there is not need to make calibrations continuously.

**Table S3.** Time required for the whole construction of the sampling and diluting paper-based biosensor and the glucose determination.

|  | | Time | |
| --- | --- | --- | --- |
| Construction of the device: Preparation of the sensing zone and the sampler and dilutor | | 30 min | |
| Glucose determination | Sampling and diluting | 30 s | 1 min 30 s |
|  | Measurement | 50 s |  |
|  | Data collection | 10 s |  |
| Total time required | | < 32 min | |

**References**

1. Li W, Qian D, Wang Q, et al (2016) Fully-drawn origami paper analytical device for electrochemical detection of glucose. Sensors Actuators, B Chem 231:230–238. https://doi.org/10.1016/j.snb.2016.03.031

2. Cinti S, Cusenza R, Moscone D, Arduini F (2018) Paper-based synthesis of Prussian Blue Nanoparticles for the development of whole blood glucose electrochemical biosensor. Talanta 187:59–64. https://doi.org/10.1016/j.talanta.2018.05.015

3. Lamas-Ardisana PJ, Martínez-Paredes G, Añorga L, Grande HJ (2018) Glucose biosensor based on disposable electrochemical paper-based transducers fully fabricated by screen-printing. Biosens Bioelectron 109:8–12. https://doi.org/10.1016/j.bios.2018.02.061

4. He XX, Chang SJ, Settu K, et al (2019) An anti-HCT-interference glucose sensor based on a fiber paper-based screen-printed carbon electrode. Sensors Actuators, B Chem 297:126763. https://doi.org/10.1016/j.snb.2019.126763

5. Cao Q, Liang B, Tu T, et al (2019) Three-dimensional paper-based microfluidic electrochemical integrated devices (3D-PMED) for wearable electrochemical glucose detection. RSC Adv 9:5674–5681. https://doi.org/10.1039/c8ra09157a

6. Cao L, Han GC, Xiao H, et al (2020) A novel 3D paper-based microfluidic electrochemical glucose biosensor based on rGO-TEPA/PB sensitive film. Anal Chim Acta 1096:34–43. https://doi.org/10.1016/j.aca.2019.10.049
